# Role of Nasal Nitric Oxide in Primary Ciliary Dyskinesia and Other Respiratory Conditions in Children

**DOI:** 10.3390/ijms242216159

**Published:** 2023-11-10

**Authors:** Salvatore Paternò, Laura Pisani, Stefania Zanconato, Valentina Agnese Ferraro, Silvia Carraro

**Affiliations:** Unit of Pediatric Allergy and Respiratory Medicine, Women’s and Children’s Health Department, University of Padova, 35128 Padova, Italy; salvatore.paterno@studenti.unipd.it (S.P.); laura.pisani@studenti.unipd.it (L.P.); stefania.zanconato@aopd.veneto.it (S.Z.); valentinaagnese.ferraro@unipd.it (V.A.F.)

**Keywords:** nasal nitric oxide (nNO), primary ciliary dyskinesia (PCD), cystic fibrosis (CF), rhinosinusitis, allergic rhinitis, children

## Abstract

Nitric oxide (NO) is produced within the airways and released with exhalation. Nasal NO (nNO) can be measured in a non-invasive way, with different devices and techniques according to the age and cooperation of the patients. Here, we conducted a narrative review of the literature to examine the relationship between nNO and some respiratory diseases with a particular focus on primary ciliary dyskinesia (PCD). A total of 115 papers were assessed, and 50 were eventually included in the review. nNO in PCD is low (below 77 nL/min), and its measurement has a clear diagnostic value when evaluated in a clinically suggestive phenotype. Many studies have evaluated the role of NO as a molecular mediator as well as the association between nNO values and genotype or ciliary function. As far as other respiratory diseases are concerned, nNO is low in chronic rhinosinusitis and cystic fibrosis, while increased values have been found in allergic rhinitis. Nonetheless, the role in the diagnosis and prognosis of these conditions has not been fully clarified.

## 1. Introduction

Nitric oxide (NO) is a gas-phase molecule synthesized by NO synthase (NOS) through the conversion of L-arginine to L-citrulline. NOS exists in three different isoenzymes, with distinct regulation and expression in the diverse tissues. Two isoenzymes, neuronal NOS (NOS1) and endothelial NOS (NOS3), are constitutively expressed in the airway epithelial cells, smooth muscle endothelial cells, and immune system cells. The third one, inducible NOS (NOS2 or iNOS), is upregulated through transcription factors activated by proinflammatory cytokines [1].

NO acts like an intracellular messenger and neurotransmitter in the upper and lower airways and plays important functions: it regulates ciliary motility and contributes to airways defense against bacterial, viral, and fungal infections, with a particular bacteriostatic action within the paranasal sinuses. In addition, it has cardiovascular functions because it increases arterial oxygen tension and decreases pulmonary vascular resistance [2].

In clinical practice, nasal and exhaled nitric oxide can be measured. Nasal nitric oxide (nNO) is produced within the nasal mucosa, mainly by the epithelial cells of paranasal sinuses, so it mirrors the upper airway status. On the other hand, fractional exhaled nitric oxide (FeNO) is produced at the level of lower airways, and it is an important biomarker of eosinophilic asthma.

While FeNO is a quite standardized parameter to be evaluated in patients with asthma, nNO is still a controversial biomarker, the pathophysiologic role of which is not fully understood yet.

The aim of the present narrative review is to provide an overview on the role of nNO in respiratory diseases, with a particular focus on the application of nNO measurement in primary ciliary dyskinesia (PCD).

## 2. Materials and Methods

In June 2023, we conducted a revision of literature consulting the PubMed database and using the following terms: (1) nasal nitric oxide AND primary ciliary dyskinesia, (2) nasal nitric oxide AND rhinosinusitis, (3) nasal nitric oxide AND allergic rhinitis, and (4) nasal nitric oxide AND cystic fibrosis. The filters applied were as follows: (1) species: human; (2) publication date: last 10 years; (3) article language: English; and (4) age: child, birth to 18 years old. After removing the duplicates, 115 manuscripts were identified. Then, two researchers separately screened all the titles and the abstracts, excluding 50 articles not mentioning nNO in the title or abstract.

**Figure 1:**
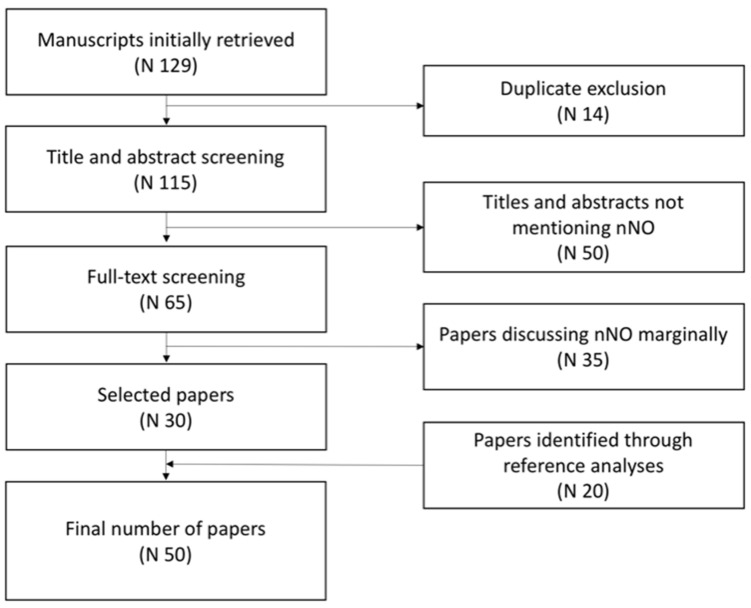
Flow chart of the paper selection process.

The researchers then performed full-text screening of the remaining 65 articles, excluding 35 papers that marginally discussed nNO. Finally, the two researchers agreed in the selection of the 30 papers. Moreover, analyzing the references of the selected papers, 20 more articles were included (Figure 1).

## 3. Results and Discussion

### 3.1. Measurement Techniques, Devices, and Age Limitations

The recommendations of the American Thoracic Society (ATS) and the European Respiratory Society (ERS) describe the possible techniques to assess nNO levels, comparing different devices on the market [3,4].

The standard device is the chemiluminescence analyzer, which exploits the reaction between NO and ozone generated by the instrument. This instantaneous reaction emits electromagnetic radiation as light (photons), which is proportional to the amount of NO molecules, sampled continuously. Advantages of this instrument are the high accuracy and the presence of a real-time display that allows the identification of the measurement endpoint: stable plateau (exhalation versus resistance and breath holding) or regular peaks (chest breathing), with no need for a fixed minimum time for sample collection.

**Figure 2:**
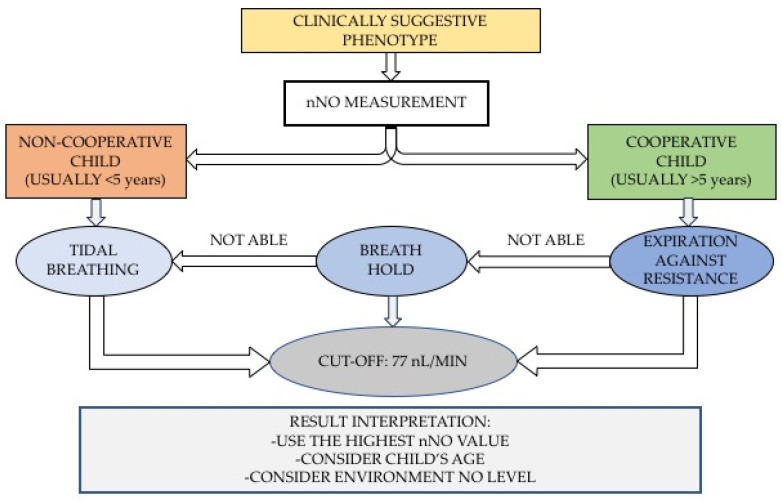
nNO measurement in PCD diagnosis: methods and result interpretation.

As a reference measurement technique, ATS and ERS recommend oral exhalation against resistance because the closure of the velum avoids contamination and dilution of nasal gas with air coming from the lower airways. This technique requires cooperation from the patient, so it is indicated in children older than 5 years. For younger children, simple breath-hold or measurement of nNO during tidal breathing can be performed. With the tidal breathing technique, the lack of velum closure implies contamination from lower respiratory tract air with greater variability of nNO values; for this reason, the results obtained with this technique should be cautiously interpreted. A recent publication of the ERS [4] summarizes how nNO should be measured according to age and compliance of the patients: a compliant patient can perform exhalation against resistance; a compliant patient, unable to complete the previous maneuver, can hold breath; a non-compliant patient can use the tidal breathing technique (Figure 2). It is recommended to take three measurements and consider the highest value.

Noteworthy, compliance and age can influence the measurement of nNO. Age, in particular, significantly affects nNO values that, in very young children, may be low even in the absence of respiratory diseases.

A recent study [5] measured nNO levels with tidal breathing on 44 healthy infants who were followed over time with six measurements between 2 weeks and 2 years of age. The authors described a longitudinal increase in the values proportional to the age. Median newborn nNO values were 46 ppb (interquartile range (IQR) 29–69 ppb), increasing at a rate of 5.4% per month up to 283 ppb (IQR 203–389 ppb) at 2 years of age. Moreover, a study conducted in newborns in the first week of life demonstrated that nNO levels were significantly lower than in older children [6].

Adams et al. [7] designed an age-based formula to estimate the expected amount of nNO according to a child’s age. This formula was based on nNO values measured in 42 infants less than 1 year old, without a history of PCD or recurrent sinopulmonary disease.

### 3.2. nNO in Primary Ciliary Dyskinesia (PCD)

PCD is a rare (1 in 20,000–60,000 live births), autosomal recessive inherited disease that causes impaired mucociliary clearance because of defective ciliary structure or impaired ciliary function [8]. These defects result in chronic mucus accumulation in the upper and lower airways, leading to chronic rhinitis, chronic wet cough, bronchiectasis and recurrent respiratory infections. Some patients also present with situs inversus. The triad, namely bronchiectasis, sinusitis, and situs inversus totalis, identified as Kartagener’s syndrome, was described for the first time in the 1930s [9].

Transmission electron microscopy (TEM), high-speed videomicroscopic analysis (HCVS), and genetic testing are the reference standard tests to diagnose PCD, although they have important drawbacks: expensive costs, prolonged time to obtain results, non-diagnostic results due to inadequate airway mucosa samples, or lack of standardization of ciliary waveform analysis [10]. Moreover, PCD diagnosis is challenging and usually delayed because symptoms are not specific, and they overlap with other conditions, such as immunodeficiency syndromes, cystic fibrosis, and recurrent respiratory infections. An international survey by Behan et al. [11] showed that 37% of patients had more than 40 specialistic evaluations due to PCD-related symptoms before being tested for PCD, and the disease is usually diagnosed at about 6 years of age in children with normal situs (slightly earlier in children with situs inversus). With PCD diagnosis being so challenging, the nNO measurement, which is rapid, economic, and reliable, quickly assumed an important role in the diagnostic process.

NO presence in exhaled breath of humans was firstly discovered by Gustafsson et al. in 1991 [12], and only 3 years later, in 1994, *Lundberg* et al. described a correlation between nNO and PCD, reporting extremely low nNO concentrations in patients with PCD [13]. Later, nNO was studied as a screening test in patients with high probability of having this disease because of a highly suggestive clinical phenotype. However, definitive diagnosis was always based on TEM, HCVS, or genetic testing. Only recently the potential of nNO not just as a screening tool but also as one of the diagnostic tests has been introduced.

One of the most recent meta-analyses performed on this topic [10] supports the role of nNO as a diagnostic tool when measured in a clinically highly suggestive population with the following criteria: (1) unexplained neonatal respiratory distress at term birth, (2) year-round wet cough starting before 6 months of age, (3) year-round nasal congestion starting before 6 months of age, and (4) organ laterality defects. The sensitivity and specificity were 0.21 and 0.99 in the presence of four criteria, 0.50 and 0.96 with three criteria, and 0.80 and 0.72 with two criteria, respectively [14].

As for the clinical characterization of patients with suspected PCD, it is worth mentioning also the score primary ciliary dyskinesia rule (PICADAR) [15]. This predictive score can be applied only in children with daily wet cough, and it investigates the presence of seven clinical characteristics: (1) full-term birth, (2) chest symptoms in the neonatal period, (3) admission to a neonatal unit, (4) situs abnormality, (5) congenital heart defect, (6) persistent perennial rhinitis, and (7) chronic ear or hearing symptoms. The PICADAR predicts the likelihood of having primary ciliary dyskinesia (PCD) with sensitivity and specificity of 0.90 and 0.75, respectively, for a cut-off score of 5 points.

Selecting a population with an appropriate clinical phenotype has a great importance because, as reported by Lucas and Walker [16], nNO alone has a low positive predictive value (42.6%), despite satisfying sensitivity and specificity rates (93.6% and 84.1%, respectively), and a high negative predictive value (99.1%). This means that a negative test (i.e., nNO levels over the cut-off levels) excludes with high probability the diagnosis of PCD, while a positive test (i.e., nNO levels below the cut-off levels) identifies more false positive than true PCD patients, and, if patients are not well selected on the base of their clinical features, the detection of a low nNO value can lead to an excessive and inappropriate use of further diagnostic tests. On the other hand, the study by Shapiro et al. [10] showed that low nNO values in the setting of an appropriate clinical phenotype is a reliable PCD diagnostic method with both sensitivity and specificity values of more than 95%.

Therefore, if the following conditions occur: (1) good cooperation of patients, (2) exclusion of CF, and (3) high clinical suspicion based on symptoms, then nNO evaluation has an equal or even better diagnostic accuracy than TEM and genetic testing.

However, diagnosis of PCD is still challenging for clinicians, and all the diagnostic instruments available need to be applied in reference centers. A 2020 Finnish study by Schultz et al. [17] showed that mutations in the Dynein Arm Heavy Chain 11 (DNAH11) gene are likely pathogenic for PCD, but they do not cause ultrastructural defects of the ciliary apparatus; therefore, in the presence of such defect, TEM would not be diagnostic alone. Also, genetic testing alone does not give enough information; in fact, identical mutations in a family showed a similar phenotype of the cilia beating pattern but significant differences in disease severity [17]. In another family, all the probands were homozygotes for the same mutation with a similar degree of disease severity, but the phenotype of the cilia beating pattern was different, ranging from stiff or static cilia to a hyperkinetic movement [17]. As for nNO measurement, the ERS guidelines [4] state that it cannot definitively confirm or rule out the diagnosis of PCD, but it helps assessing the probability of PCD when used together with the other tests. Therefore, only applying all the reference testing methods we can make a complete clinical and instrumental evaluation of a patient with suspected PCD.

#### 3.2.1. nNO Cut-Off Levels for PCD

In 2013, Leigh et al. [18] reported nNO levels measured in 143 PCD patients, with confirmed ultrastructural defects detected by TEM, comparing the values with those measured in subjects with other lung diseases and in healthy controls. The average value in PCD subjects was 20.7 ± 24.1 nL/min (standard deviation) with a range from 1.5 to 207.3 nL/min. These data rarely overlapped with the nNO values of healthy control subjects (304.6 ± 118.8, ranging from 125.5 to 867.0 nL/min), patients with asthma (267.8 ± 103.2, ranging from 125.0 to 589.7 nL/min), or patients with chronic obstructive pulmonary disease (223.7 ± 87.1, ranging from 109.7 to 449.1 nL/min). The only overlapping data were with nNO levels in patients with cystic fibrosis (134.0 ± 73.5, ranging from 15.6 to 386.1 nL/min).

The disease-specific nNO cut-off value was then established at 77 nL/min, with sensitivity of 0.98 and specificity > 0.999.

The cut-off for PCD is the same regardless of the technique used. The meta-analysis by Collins et al. [19] compared data from three studies that evaluated nNO values in patients with PCD [20,21,22], finding no difference between the measurement performed with tidal breathing and those against velum closure (21.6 nL/min versus 19.8 nL/min).

Nonetheless, the ERS document [4] mentions the possibility of considering lower cut-offs when the measure is performed in very young children during tidal breathing: 30 nL/min for children 1–2 years old and 44 nL/min for children older than 2. Noteworthy, these cut-offs have been suggested by small single-center studies, and they need to be validated in multicenter studies.

When considering the value of nNO measures, some possible confounding factors should be considered.

Rybnikar et al. [23] performed nNO measurements in 48 non-allergic patients aged 5 to 18 years with chronic symptoms of nasal obstruction and surgical indication for adenoidectomy. The authors found that, before adenoidectomy, average nNO values were inversely related to the degree of hypertrophy, so a major hypertrophy reduces nNO levels. As a consequence, applying the standard cut-off level, 7 of these patients (14.6%) would have been classified as PCD, while after adenoidectomy, none of them had an nNO level below the cut-off. Therefore, if adenoid hypertrophy is suspected, nNO is not a reliable diagnostic tool for PCD.

In addition, environment NO levels should be considered when interpreting nNO values. In fact, NO environment concentrations greater than 20 ppb can affect nNO measurement [4].

#### 3.2.2. Relationship between nNO, Genotypes, and Ciliary Function: A Molecular Focus

The mechanism by which nNO is reduced in PCD has yet to be elucidated. Respiratory epithelial cells have been studied to determine their ability to synthesize NO, and the cells obtained from PCD patients seem to produce a similar amount of NO amounts compared with those from non-PCD patients [24].

Some interesting hypotheses were collected by Walker et al. in their review [25]. nNO reduction can be explained by increased NO degradation, either within the cell or externally (in the viscous mucus layer or by denitrifying bacteria). It has been also hypothesized that NO is sequestered in the upper airways within the obstructed sinuses or, alternatively, that NO storage capacity in PCD patients is limited. However, none of these hypotheses has been definitively proved.

A number of studies investigated the relationship between the levels of nNO and the type of ultrastructural or genetic defect. In a recent study by Legendre et al. [26], 73 children with PCD were retrospectively studied. They were divided into three groups, according to their ciliary motility (motile, immotile, and mixture); then, the relationship between phenotypes and pathogenic mutations was explored. They found that higher nNO levels were more frequently associated with mild genotypes or hypomorphic mutations (missense, single amino acid deletion, or moderate splicing mutations), while children with severe mutations (biallelic truncating mutations) typically registered a lower nNO level. For example, patients with immotile cilia had a mean nNO value of 16 nL/min (range 10–23 nL/min), patients with dyskinetic motile and immotile cilia had a mean nNO value of 23 nL/min (range 17–56 nL/min), and patients with dyskinetic but motile cilia had a mean nNO value of 78 nL/min (range of 45–93 nL/min), borderline with cut-off values.

In another study by Knowles et al. [27], normal nNO levels were measured in PCD patients with specific genetic alterations, like Radial Spoke Head Component 1 (*RSPH1*) mutations, that seem associated with residual ciliary function and a milder phenotype. In fact, cilia from patients with RSPH1 mutations had normal beat frequency but an abnormal circular beat pattern.

Also, a recent study by Pifferi et al. [28] evaluated longitudinally nNO levels in PCD patients. In this prospective study, patients with PCD were divided in two groups: (1) patients with specific gene mutations (i.e., biallelic mutations in CCDC39 and CCDC40) and ultrastructural abnormalities (i.e., inner dynein arm (IDA) and microtubular disorganization (MTD)) usually associated with worse pulmonary outcome over time (measured through the deterioration of spirometry and lung volumes); and (2) patients without those genetic and ultrastructural alterations and therefore likely to have a better lung function prognosis [29,30]. The ultrastructural group with the worst lung function evolution (IDA and MTD) showed a decline in nNO with age, and the genetic group with worst lung function evolution (CCDC39 and CCDC40) showed the steepest decline in nNO over time compared with all the other genotypes. The study also showed that patients with higher nNO had lower prevalence of positive bacterial isolate from the lower respiratory tract.

Therefore, it seems that nNO can also have a prognostic role in PCD, although it is not clear yet whether lower nNO levels are directly related to a poorer prognosis or whether are the associated ultrastructural and genetic alterations or the infections that lead to worse disease course. Therefore, studies are needed to investigate the possible role of therapeutic strategies aimed at increasing upper airway NO production, as already trialed with inhaled and oral L-arginine supplementation in patients with cystic fibrosis [31].

In this regard, Kouis et al. [32] hypothesized that, if L-arginine acts like a nitrogen donor for NO production and NO mediates ciliary motility, increasing L-arginine concentration can stimulate ciliary function. In fact, they were able to increase ciliary beat frequency (CBF) by increasing L-arginine concentration in a suspension of airway ciliated epithelial cells obtained from nasal biopsies. This result suggested a possible role for L-arginine as a potential stimulator of mucociliary clearance in congenital ciliary disorders with residual motility, but further studies are needed to confirm these findings.

Beside its involvement in PCD pathophysiology, NO seems to play a role as a molecular mediator also in other processes involving cilia.

Pifferi et al. [33] investigated in PCD patients the sense of smell, in which cilia are involved. They found a worse olfactory function in PCD patients with major ultrastructural abnormalities and lower nNO levels. On the other hand, olfactory sensation was less compromised in patients with ciliary defects but with a relatively preserved ciliary function. They hypothesized a role of NO in the neural transmission of olfactory stimuli. In an animal model, during olfactory stimuli transmission, NO production induced a cGMP increase within the entire neuron [34]. Moreover, increased hippocampal and olfactory bulb neurogenesis is associated with increased regional endothelial nitric oxide synthase (NOS3) expression [35].

Another function of cilia is involved in left–right patterning during embryogenesis, and a dysfunction can lead to possible cardiovascular anomalies. Assessing ciliary function in patients with heterotaxy, Nakhleh et al. [36] found a significant risk for respiratory diseases, high prevalence of alterations in PCD genes, and reduced levels of nNO, often below the PCD cut-off.

Low nNO was also found in patients with non-heterotaxic congenital heart disease with respiratory post-surgical complications. Stewart et al. [37] hypothesized that low nNO can reduce hemodynamic function, and, therefore, pre-operative nNO screening may guide early interventions to reduce pulmonary morbidities.

Finally, in patients with transposition of the great arteries (TGA), high prevalence of abnormal ciliary motion and low nNO levels were found. However, ciliary dysfunction was not correlated with the TGA type, and respiratory symptoms were not significantly connected with a low nNO value [38]. 

### 3.3. Applications of nNO in Other Respiratory Inflammatory Diseases

As previously discussed, the diagnostic role of nNO is well studied in PCD, while its diagnostic or prognostic role in other upper and lower respiratory diseases is still debated.

Previous studies found that patients with chronic rhinosinusitis have lower levels of nNO than the control group, while patients with allergic rhinitis have higher nNO levels than the healthy population [1] (Table 1).

Chronic rhinosinusitis is characterized by chronic inflammation of paranasal sinuses; it presents with running nose, nasal obstruction, pain when pressing forehead and maxillary area, and loss of smell; and it can be associated with nasal polyposis. Diagnosis is primarily based on clinical symptoms and imaging techniques, but nNO measurement can be a non-invasive, safe, and inexpensive tool (features that are particularly useful in the pediatric population). Since nNO is mostly produced in paranasal sinus mucosa, reduction in nNO levels in chronic rhinosinusitis was classically explained by a chronic damage of epithelial cells, the main producer of NO in upper airways, and by the mechanical obstruction of sinus ostium by mucus secretions and polyps [1].

Yoshida et al. [2] measured nNO in patients with chronic rhinosinusitis and nasal polyposis before and after polyps removal surgery. After endoscopic sinus surgery (ESS), nNO levels were not increased as expected, indicating that nNO measurement is not so strictly associated with anatomical obstruction, but it is rather the consequence of wellness and integrity of epithelial cells of paranasal sinuses. In fact, 6 months after surgery, nNO levels showed an increasing trend, although not statistically significant, as if nasal mucosa was gradually recovering in a re-epithelialization process. In an intact nasal mucosa, the tissue plasminogen activator (t-PA) is constitutively expressed in nasal epithelial cells, and it converts plasminogen to plasmin, which has a fibrinolytic action. In addition, the t-PA binds to a cell membrane receptor called low-density-lipoprotein receptor-related protein-1 (LRP-1), activating the receptor tyrosine phosphorylation and an intracellular signal transduction through the NF-k-B signaling pathway, that finally induces the expression of inducible NO synthase (iNOS) and thus NO production.

In eosinophilic chronic rhinosinusitis, nasal mucosa is infiltrated with eosinophils, which induce a Th2-type of inflammatory response, with production of inflammatory cytokines that downregulate t-PA expression, with lower NO production. In addition, during wound healing, NO is responsible for replacing the fibrin matrix with collagen produced by fibroblast. Therefore, if NO production is reduced, collagen production is downregulated and fibrin removal inhibited, causing a prolonged inflammation and edema in the nasal mucosa, the perfect environment to develop recurrent polyposis [2].

Describing these molecular mechanisms, Yoshida et al. [2] tried to explain the reduced levels of nNO in chronic rhinosinusitis, which can be both the cause or the consequence of chronic inflammation, and suggested that nNO measurement can be a marker of the severity of the disease. They also suggested a possible therapeutic approach based on nasal NO induction against nose and sinuses inflammation, but further studies on NO pathogenesis are needed.

As previously stated, while pediatric patients with chronic rhinosinusitis have reduced nNO levels, patients with allergic rhinitis show an upregulation of nNO levels [39,40], although nNO has a controversial role as a diagnostic tool in this respiratory condition.

In the study of Sutiratanachai et al. [41], 48 children with house dust mite (HDM)-induced allergic rhinitis were tested for nNO levels, finding a positive correlation between higher nNO levels and the degree of HDM sensitization.

Allergic rhinitis is an inflammatory condition of the upper respiratory airway due to an allergen exposition, which causes a Th2-type inflammatory response, with eosinophilic inflammation and release of histamine and inflammatory cytokines. Allergic airway inflammation promotes the hyperactivity of NOS2 and then aggravates the production of NO concentration in the airway [41].

Patients with allergic rhinitis present with nasal itching, runny nose, and nasal obstruction. It is the most common allergic disease in the adult and pediatric population and, although it is not a severe condition, it impairs the quality of life and, if underdiagnosed or not adequately treated, can also progress to lower respiratory diseases [42].

An interesting study by Ferrante et al. [43] pointed out the role of nNO as a significant biomarker of therapeutic control with intranasal steroids in allergic rhinitis. They conducted a 3-week, randomized, double-blind study in 48 children with perennial allergic rhinitis who received nasal budesonide or nasal saline solution. Children treated with nasal budesonide showed a significant reduction in nNO levels in comparison to the placebo group. Other studies found the same reduction in nNO in adults and children with allergic rhinitis treated with nasal corticosteroids [44]. At a molecular level, the corticosteroid effect is explained by the inhibition of the transcription nuclear factor (NF-kB) that normally mediates the transcription of the iNOS gene [45].

Lately, nNO in allergic rhinitis patients is also acquiring a prognostic role. A recent study by Wen et al. [46] applied the measurement of nNO levels and the NOS2 serum level to predict the clinical efficacy of subcutaneous immunotherapy (SCIT) in allergic rhinitis pediatric patients. The 160 study participants were divided into 40 healthy controls and 120 patients with allergic rhinitis. nNO were measured in both groups and appeared to be significantly higher in the second group. The allergic rhinitis group underwent 1-year SCIT and was then divided into responders and non-responders, with the former showing higher levels of nNO and serum NOS2 at baseline.

In fact, SCIT inhibits eosinophilic inflammation and moves the inflammatory process toward a Th1-type inflammatory response. High nNO levels in patients with allergic rhinitis are thought to be proportional to the intensity of eosinophilic inflammation, and, as a result, a higher nNO level means a bigger target for SCIT. This study pointed out that the combined use of nNO and serum NOS2 might serve as a reliable and useful method for predicting the clinical outcome of SCIT in patients with allergic rhinitis.

A previous study by Parisi et al. [47] made a similar evaluation to identify the efficacy predictors of sublingual immunotherapy treatment (SLIT) in 34 pediatric patients with allergic rhinitis. Their data, beside confirming the effectiveness of SLIT from a clinical perspective, identified nNO and nasal cytology as predictive biomarkers of treatment efficacy in the short term.

While NO concentration in airways is usually high in chronic inflammatory diseases (allergic rhinitis, bronchiectasis, etc.) because of the induction of NO synthase by inflammatory cytokines such as TNF-a, IL-1b, and interferon-c [48], cystic fibrosis (CF) represents an exception since CF patients usually have normal or even reduced nNO levels [49] (Table 1).

De Winter–de Groot et al. [50] analyzed this counterintuitive finding and formulated some possible explanations: patients with CF may have a poor nutritional status that can lead to the lack of L-arginine, the substrate of NO synthase. In addition, thick mucus lining in the airways might prevent the diffusion of NO into the gaseous phase or might obstruct nasal sinuses. Finally, NO synthase might present polymorphisms related to the CF genotype that can influence NO production.

They conducted a cross-sectional study comparing data from a population of 95 children and adolescents with CF, and they found that patients with CF and nasal polyposis, compared with those without polyposis, had lower nNO levels, which raised after functional endoscopic sinus surgery (FESS), supporting the hypothesis of mechanical obstruction as one of the causes of a reduced nNO level. However, after surgery, nNO levels did not reach normal levels, so the obstruction cannot be the only explanation of the low nNO levels in CF; this result is in line with the study of Yoshida et al. [2] on the role of nasal mucosa damage with respect to low nNO levels in patients with rhinosinusitis and polyposis.

Kirihene et al. [51], on the contrary, found that surgical enlargement of the maxillary sinus ostium was associated with lower nNO levels, probably because enlarging the sinus can increase the flow through it, and nNO measurements are inversely proportional to flow: a higher or more turbulent flow might produce a lower nNO value.

As regard the other hypothesis suggested by De Winter–de Groot et al. [50], no correlation between NO levels and the nutritional status emerged, as well as between nNO levels and the CF genotype.

Another study by Michl et al. [52] focused on the relationship between nNO and microbiological pathogens in patients with CF, finding a negative correlation between nasal NO and biochemical signs of infections. On one hand, reduced nNO means reduced bacteriostatic activity, which facilitates upper airway infection; on the other hand, reduced nNO can be the consequence of a primarily acute exacerbation of CF, with increased and thicker secretions that can retain NO metabolites in the mucus and NO possibly being denitrified by the increased number of bacteria within the paranasal sinuses.

In addition, in children with CF and polyps, after FESS, higher nNO levels and fewer S. aureus-positive cultures were detected, supporting the host defense function of NO in the upper airways [50]. In summary, it is generally acknowledged that the upper airways in CF patients have an impaired defense mechanism that facilitates chronic pathogen colonization, but further studies on the role of nNO need to be performed.

## 4. Conclusions

In this narrative review, we tried to summarize all the most recent and interesting findings about the role of nNO in respiratory diseases in children. nNO evaluation in patients depends on age and cooperation, so it is important to choose the right device and measurement technique, with oral exhalation against resistance being the recommended technique.

nNO has an important and validated role in PCD diagnosis, when evaluated in a clinically suggestive phenotype. The nNO cut-off currently used is 77 nL/min, with PCD patients showing nNO levels below the cut-off.

As far as other respiratory diseases are concerned, nNO is low in patients with chronic rhinosinusitis, probably due to a chronically damaged mucosa. Patients with allergic rhinitis show, on the other hand, an increased nNO value, probably due to the Th2-type inflammation. Finally, patients with cystic fibrosis present low levels of nNO, and the reduced levels of this biomarker are associated with higher prevalence of infections.

In respiratory diseases with low nNO levels, a novel and challenging therapeutic strategy can be increasing NO production to achieve the beneficial effects of NO with respect to ciliary movements and antimicrobial action.

In conclusion, nNO is a molecular mediator with not only a well-documented role in PCD but also a potential importance in the characterization of other respiratory diseases. Further studies are needed to evaluate the NO role as therapeutic target strategy in debilitating respiratory diseases like PCD and CF.

## Figures and Tables

**Table 1:** nNO in pediatric respiratory diseases: detected levels, suggested mechanisms, and possible clinical use.

Disease	nNO Levels	Suggested Mechanisms	Clinical Use
PCD	Low	Increased NO degradation Reduced NO storage capacity	PCD diagnosis in a clinically suggestive patient
Rhinosinusitis	Low	Chronic damage of epithelial cells Mechanical obstruction of sinus ostium due to mucus secretions and polyps	Possible marker of severity
Allergic rhinitis	High	Inflammatory cytokines promote NOS2 activity, increasing NO production	Possible prognostic role in assessing immunotherapy response
Cystic fibrosis	Normal or low	Thick mucus might prevent diffusion of NO into the gaseous phase Obstructed nasal sinuses might prevent NO diffusion	Reduced levels can be associated with higher prevalence of infections

## Data Availability

No new data were created for the manuscript.
